# Repeated mapping of cortical language sites by preoperative navigated transcranial magnetic stimulation compared to repeated intraoperative DCS mapping in awake craniotomy

**DOI:** 10.1186/1471-2202-15-20

**Published:** 2014-01-30

**Authors:** Sandro M Krieg, Nico Sollmann, Theresa Hauck, Sebastian Ille, Bernhard Meyer, Florian Ringel

**Affiliations:** 1 Department of Neurosurgery, Klinikum rechts der Isar, Technische Universität München, Ismaninger Straße 22, Munich, 81675, Germany

**Keywords:** Preoperative mapping, Broca’s area, Tumor, Transcranial magnetic stimulation, Navigated brain stimulation

## Abstract

**Background:**

Repetitive navigated transcranial magnetic stimulation (rTMS) was recently described for mapping of human language areas. However, its capability of detecting language plasticity in brain tumor patients was not proven up to now. Thus, this study was designed to evaluate such data in order to compare rTMS language mapping to language mapping during repeated awake surgery during follow-up in patients suffering from language-eloquent gliomas.

**Methods:**

Three right-handed patients with left-sided gliomas (2 opercular glioblastomas, 1 astrocytoma WHO grade III of the angular gyrus) underwent preoperative language mapping by rTMS as well as intraoperative language mapping provided via direct cortical stimulation (DCS) for initial as well as for repeated Resection 7, 10, and 15 months later.

**Results:**

Overall, preoperative rTMS was able to elicit clear language errors in all mappings. A good correlation between initial rTMS and DCS results was observed. As a consequence of brain plasticity, initial DCS and rTMS findings only corresponded with the results obtained during the second examination in one out of three patients thus suggesting changes of language organization in two of our three patients.

**Conclusions:**

This report points out the usefulness but also the limitations of preoperative rTMS language mapping to detect plastic changes in language function or for long-term follow-up prior to DCS even in recurrent gliomas. However, DCS still has to be regarded as gold standard.

## Background

The restriction of language function to the classical Broca’s or Wernicke’s area is not compatible anymore [1-3]. Shaping cortical reorganization due to tumor-induced impairment of normal function was shown for cortical but also for subcortical structures and pathways [4].

Therefore, it is important to detect individual language associated sites prior to surgery of gliomas in perisylvian brain regions. Giussani et al. performed a review on the current studies comparing functional magnetic resonance imaging (fMRI) with intraoperative language mapping by direct cortical stimulation (DCS). They reported that sensitivity and specificity were ranging from 59% to 100% and from 0% to 97%, which lead to the conclusion that fMRI can not be considered as an alternative to DCS with the current technique. Thus, there is still a need for a reliable preoperative mapping of language eloquent cortical regions [5]. Just recently, navigated transcranial magnetic stimulation (nTMS) was described as a method for preoperative mapping of motor and language eloquent cortical regions [6-10]. Yet, there is still no evidence whether nTMS is able to detect language plasticity in brain tumor patients prior to surgery.

This report illustrates the first clinical series of three right-handed patients suffering from language eloquent brain tumors of the left hemisphere. All patients were mapped for language eloquent cortical regions preoperatively by repetitive nTMS (rTMS) and intraoperatively during awake surgery by DCS prior to initial and recurrent tumor resection. By comparing the results of both mappings in every individual case, this study provides a comparison between language mapping by rTMS and DCS during repeated awake surgery in a follow-up series with focus on language plasticity.

## Methods

### Patients

**Figure 1:**
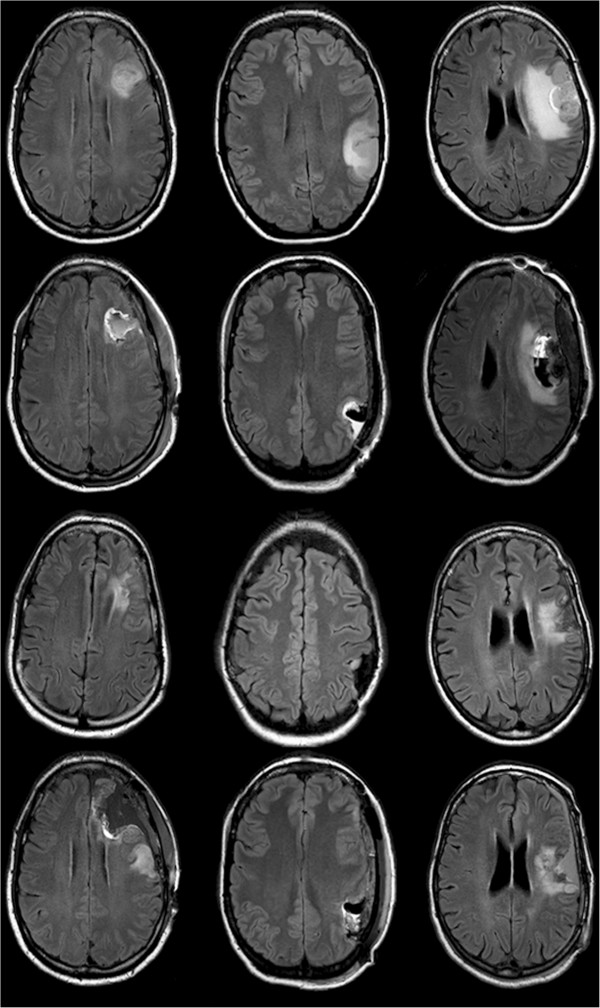
**Pre- and postoperative MRI scans.** Upper row: Initial tumor of patient 1–3; 2nd row: postoperative MRI of patient 1–3 shows resection after first surgery; 3rd row: recurrent tumor of patient 1–3; lower row: postoperative MRI of patient 1–3 shows resection after second surgery.

Three right-handed patients, suffering from left-sided opercular glioblastomas in two cases (47 years old male and 51 year old female) and from a left-sided astrocytoma WHO grade III of the angular gyrus (29 year old female) (Figure 1), initially presented with focal seizures. Each patient was a German native speaker without any other primary language. Table 1 provides an overview of the enrolled patients. The day before surgery, the patients underwent navigational MRI followed by preoperative language mapping by rTMS. Intraoperatively, DCS mapping for language-positive sites was performed during awake surgery as previously described [11]. This setup for cortical language mapping was performed for initial as well as for repeated tumor resection in all three patients. There was no neoadjuvant therapy prior to the first surgery in all cases. Yet, both GBM patients received radiotherapy with 60 Gy for 6 weeks and 6 cycles of temozolomide (TMZ) according to the Stupp setup between both surgeries. The patient with astrocytoma WHO grade III only received TMZ between both surgeries. Indication for repeated awake surgery was done by an interdisciplinary tumor conference involving neurosurgeons, neurooncologist, neuroradiologists, and radiation oncologists. Between the first and repeated surgery, there was an interval of 7, 10, and 15 months (Table 1).

**Table 1:** Patient characteristics

	**Patient 1**		**Patient 2**		**Patient 3**	
	**1st surgery**	**2nd surgery**	**1st surgery**	**2nd surgery**	**1st surgery**	**2nd surgery**
**Age (years)**	47	48	29	30	51	52
**Gender**	Male		Female		Female	
**Years of education**	10		10		19	
**Tumor entity**	glioblastoma WHO grade IV	glioblastoma WHO grade IV	astrocytoma WHO grade III	astrocytoma WHO grade III	glioblastoma WHO grade IV	glioblastoma WHO grade IV
**Tumor location**	opIFG	opIFG	anG	anG	opIFG	opIFG
**Tumor volume (cm**^**3**^**)**	20.6	21.7	65.5	6.7	195.4	45.3
**Time between surgeries (months)**	7		15		10	
**Adjuvant therapy before surgery**	none	RTx & TMZ	none	TMZ	none	RTx & TMZ
**Correct baseline pictures (out of 131)**	104 (79%)	102 (78%)	101 (77%)	98 (75%)	81 (62%)	98 (75%)
**RMT (% output)**	30	30	34	35	28	33
**Mapping intensity (% of MT)**	110	100	100	100	100	100
**Most comfortable frequency**	5 Hz/5	7 Hz/5	5 Hz/5	5 Hz/5	5 Hz/5	7 Hz/7
**Pain during stimulation (VAS): convexity**	3	4	2	4	1	7
**Pain during stimulation (VAS): temporal**	6	7	7	6	4	7
**Number of errors left hemisphere**	119	62	151	135	151	83
**Number of stimulations left hemisphere**	750	390	675	405	641	258
**Error rate left hemisphere**	15.9%	15.9%	22.4%	33.3%	23.6%	32.2%
**no response**	3.3%	6.2%	13.3%	8.6%	2.8%	8.9%
**performance**	2.5%	1.3%	2.5%	7.4%	8.6%	9.3%
**hesitation**	9.7%	7.4%	5.6%	16.0%	9.5%	13.6%
**neologism**	0.3%	0.0%	0.7%	0.7%	2.7%	0.0%
**phonological error**	0.0%	0.5%	0.0%	0.0%	0.0%	0.0%
**semantic error**	0.0%	0.5%	0.1%	0.5%	0.0%	0.4%
**Preoperative aphasia**	0	0	0	1A	1A	2A
**Postoperative aphasia (5th day after surgery)**	0	0	1B	1A	2A	2A
**Postoperative aphasia (3 months after surgery)**	0	0	0	1A	0	2A
**Extent of tumor resection**	Complete	Complete	Complete	Complete	Complete	Partial

This table shows the characteristics of all patients including pre- and intraoperative findings. Aphasia scale: 0 (no aphasia), 1 (mild aphasia with unremarkable communication), 2 (slightly impaired communication), 3 points (severe grade of aphasia); motoric aphasia = A, sensory aphasia = B. Moreover, the extent of tumor resection is presented. RMT = resting motor threshold (% stimulator output), VAS = visual analogue scale, RTx = radiotherapy, TMZ = Temozolomide.

### Ethical standard

The study is in accordance with ethical standards of the local institutional review board (registration number: 2793/10) and the Declaration of Helsinki. Informed consent was obtained prior to every rTMS examination from every patient including consent for publication of individual clinical details.

### Preoperative MRI

All patients underwent MRI prior to each resection and during follow-up every 3 months by a 3 Tesla magnetic resonance scanner (Achieva 3 T, Philips Medical Systems, The Netherlands B. V.) as described before [10]. In short, the scanning protocol consisted of a T2 FLAIR (TR/TE 12000/140, inversion time of 2500 ms, 30 slices with 1 mm gap, voxel size 0.9 × 0.9 × 4 mm^2^, 3 min acquisition time) and a 3D gradient echo sequence (TR/TE 9/4, 1 mm^2^ isovoxel). For anatomical co-registration the patient received intravenous contrast agent (0.1 mmol/kg body weight gadopentetate dimeglumine, Magnograf, Marotrast GmbH, Jena, Germany).

### Preoperative rTMS

**Figure 2:**
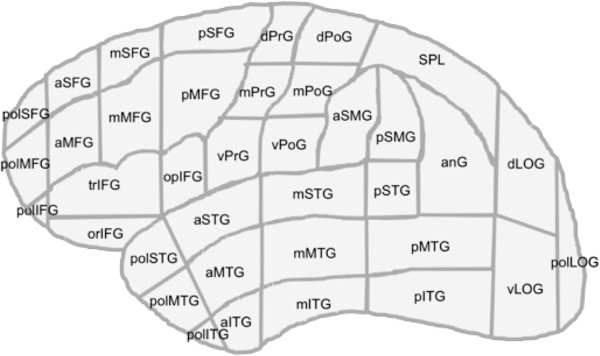
**Cortical parcellation system (CPS).** This graph shows the location of all regions of the CPS.

The rTMS system (eXimia 4.3, Nexstim, Helsinki, Finland) consists of a computer panel and a magnetic stimulator with a biphasic figure-of-eight coil [12-14]. As outlined in an earlier report, language eloquent areas were identified via functional testing by an object-naming task and rTMS with 5 to 7 Hz, 5 to 7 pulses according to a virtual lesion model [7,10,15]. The term ‘virtual lesion’ based on the theory on causing a transient functional underactivity during the time of stimulation. Naming errors elicited by rTMS were counted and categorized. There were six main categories: no responses, performance errors, hesitations, neologisms, phonological, and semantic errors. For every single error category as well as for all errors, an error rate was defined as the ratio of induced errors to the number of applied rTMS trains. The number of stimulations per patient mainly relied on the general health status of our tumor patients, their grade of fatigue, and their ability to focus on the examination, which can be quite different in brain tumor patients. The cortical parcellation system (CPS) was used for further anatomy-related data analysis and visualization [16,17]. This system parcellates the cortex into 37 regions with special regard to anatomical brain structures (Figure 2, Table 2).

**Table 2:** Cortical parcellation system (CPS)

**Abbreviation**	**Anatomy**
aITG	Anterior inferior temporal gyrus
aMFG	Anterior middle frontal gyrus
aMTG	Anterior middle temporal gyrus
anG	Angular gyrus
aSFG	Anterior superior frontal gyrus
aSMG	Anterior supramarginal gyrus
aSTG	Anterior superior temporal gyrus
dLOG	Dorsal lateral occipital gyrus
dPoG	Dorsal post-central gyrus
dPrG	Dorsal pre-central gyrus
mITG	Middle inferior temporal gyrus
mMFG	Middle middle frontal gyrus
mMTG	Middle middle temporal gyrus
mPoG	Middle post-central gyrus
mPrG	Middle pre-central gyrus
mSFG	Middle superior frontal gyrus
mSTG	Middle superior temporal gyrus
opIFG	Opercular inferior frontal gyrus
orIFG	Orbital part of the inferior frontal gyrus
pITG	Posterior inferior temporal gyrus
pMFG	Posterior middle frontal gyrus
pMTG	Posterior middle temporal gyrus
polIFG	Polar inferior frontal gyrus
polITG	Polar inferior temporal gyrus
polLOG	Polar lateral occipital gyrus
polMFG	Polar middle frontal gyrus
polMTG	Polar middle temporal gyrus
polSFG	Polar superior frontal gyrus
polSTG	Polar superior temporal gyrus
pSFG	Posterior superior frontal gyrus
pSMG	Posterior supramarginal gyrus
pSTG	Posterior superior temporal gyrus
SPL	Superior parietal lobe
trIFG	Triangular inferior frontal gyrus
vLOG	Ventral lateral occipital gyrus
vPoG	Ventral post-central gyrus
vPrG	Ventral pre-central gyrus

This table outlines the definition of all regions of the CPS.

Moreover, local pain during the rTMS sessions was measured via a visual analogue scale (VAS). Therefore, every subject was asked to rate the discomfort or pain from 0 (no pain) to 10 points (maximum pain) separately for convexity (defined as the lateral, dorsal, rostral, and cranial surface of the brain without the region covered by the temporal muscle) and temporal regions.

### DCS mapping during repeated awake surgery

**Figure 3:**
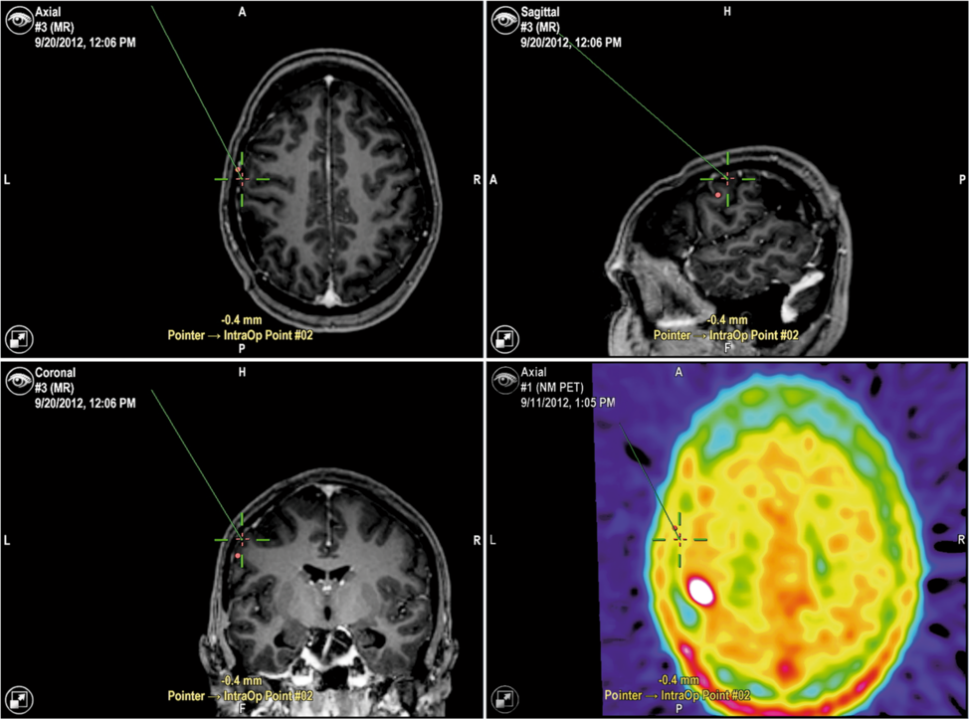
**Data transfer.** The screenshot shows the data transfer to the neuronavigation system of intraoperative DCS-positive points of patient 2 (BrainLAB Curve, BrainLAB AG, Feldkirchen, Germany).

After craniotomy, DCS was performed with a bipolar electrode (Inomed Medizintechnik, Emmendingen, Germany) with an intensity of 4–6 mA square-wave pulses with a duration of 4 seconds and frequency of 60 Hz. To detect epileptic seizures and monitor possible inhibition of adjacent cortical areas, a surface electroencephalogram (bandpass 10 Hz – 1.5 kHz) was recorded with a cortical grid electrode. An object-naming task was used during cortical mapping since as its disturbance is a common feature shared by all forms of aphasia [18]. Analogue to preoperative rTMS, all sites were stimulated three times while the patients were asked to name visually presented objects. Yet, the used pictures differed between DCS and rTMS in order to avoid learning effect. However, both picture sets showed the same or comparable objects. The patients were advised to name objects in combination with the matrix sentence “This is a…” during cortical stimulation time-locked to object presentation. All identified sites were marked with tags and transferred to the neuronavigation system (BrainLAB Vector Vision Sky and BrainLAB Curve, Brain LAB, Feldkirchen, Germany; Figure 3). In order to rule out that brain shift or any dislocation of cortical regions did affect our results, the neuronavigational method was combined with standard anatomical identification by considering gyral structure and cortical veins as references [11,19].

### Postoperative assessment

The neurological status of each patient was assessed at the first postoperative day and during postoperative follow-up. Additionally, the grade of aphasia was evaluated by the usage of a special aphasia scale, which simply uses spontaneous speech and impairment of normal verbal communication. The scale ranged from 0 (no aphasia), 1 (mild aphasia with unremarkable communication), 2 (slightly impaired communication) to 3 points (severe grade of aphasia). If there was a nonfluent aphasia, the letter “A” is added to the individual grade, whereas “B” stood for a fluent aphasia. Testing was performed before surgery, on the 5th postoperative day as well as three months after the first and the second surgery. Moreover, each patient underwent MR imaging the day after surgery (Figure 1). All patients underwent adjuvant therapy before re-resection (Table 1).

### Data analysis

To determine whether an individual brain region gave rise to language deficits during rTMS, the following definitions for region positivity and negativity were used: Positive brain region: A region was considered to give rise to language deficits if any of the trains delivered to the region elicited naming errors, regardless of the error type; Negative brain region: A brain region was considered not to give rise to language deficits if the region had been stimulated with at least one stimulation train and no language deficits of any error type were generated. Following these rules and regarding the intraoperative DCS result as the “ground truth” for each anatomical region, the rTMS results were labeled as true positive, true negative, false positive or false negative (TP, TN, FP, FN). Thereafter, the sensitivity and specificity values were calculated. This comparison could be made for each CPS region studied with intraoperative DCS.

## Results

**Figure 4:**
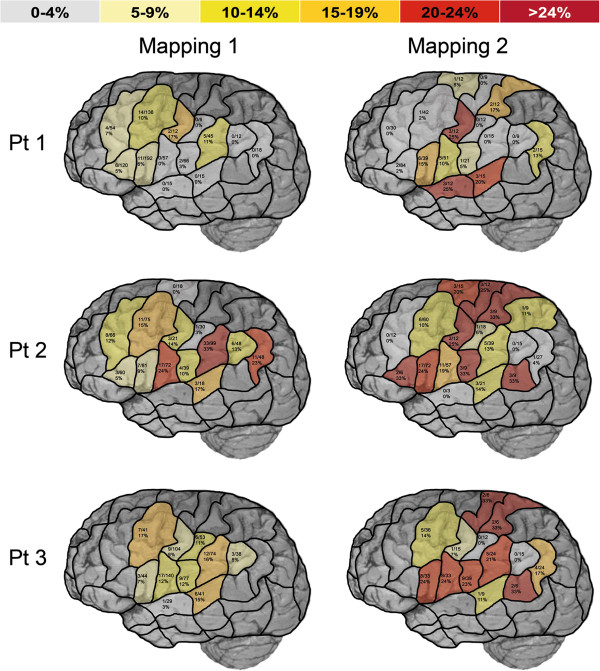
**Language mapping by rTMS.** This surface view of the human cortex shows the results of preoperative mapping of language eloquent areas by rTMS and an object naming task presented with the cortical parcellation system (CPS). Left column represents the first mapping, the right column outlines the mapping prior to the second surgery. Row 1–3 represent patient 1–3. For every single CPS region, a combined error rate for no responses and performance errors was defined as the ratio of induced no response and performance errors to the number of applied rTMS trains.

**Figure 5:**
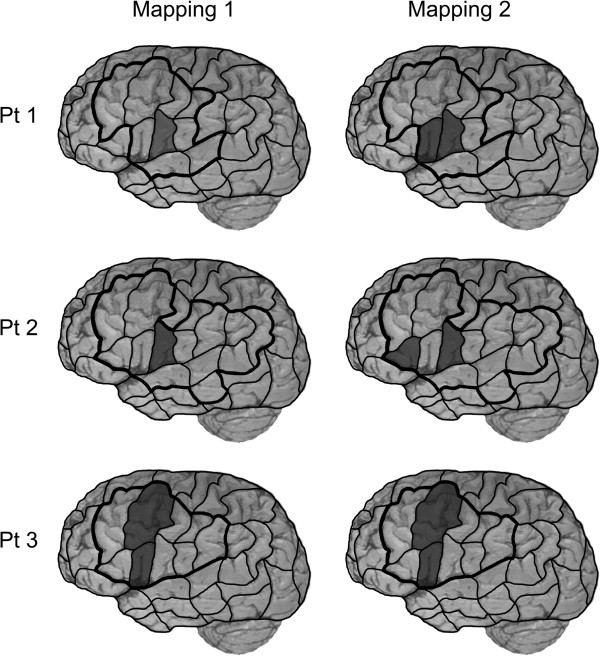
**DCS mapping.** This surface view of the human cortex visualizes language-positive regions during intraoperative DCS language mapping using an object-naming task presented with the cortical parcellation system (CPS). Left column represents the first, the right column outlines the second awake surgery. Rows 1–3 represent patient 1–3. Dark areas = DCS positive CPS regions; black line = craniotomy limits.

Table 1 and Figure 4 give an overview on mapping parameters and the distribution of no responses and performance errors separated into the different patients and mappings. Receiver operating characteristics for the combined error group are shown in Table 3. Moreover, Figure 5 provides a graphical overview on the distribution of language-positive DCS spots for all patients.

**Table 3:** Receiver operating characteristics

**Definition of rTMS-positive**	**Error rate**				
	**≥5%**	**≥10%**	**≥15%**	**≥20%**	**≥25%**
PPV	21%	24%	29%	21%	17%
NPV	93%	91%	89%	83%	82%
Sensitivity	90%	80%	60%	30%	10%
Specificity	28%	46%	67%	76%	89%

ROC on the correlation of repetitive navigated transcranial magnetic stimulation (rTMS) to direct cortical stimulation (DCS) for the combined group of no responses and performance errors across all mappings of this report. PPV = positive predictive value, NPV = negative predictive value. DCS represents the ground truth. In the second row information is giving at which percentage of error rate a CPS region is defined as rTMS positive. According to this threshold level, we observe different patterns of correlation with intraoperative DCS.

### Patient 1 – first mapping

#### Preoperative rTMS

During the preoperative rTMS mapping of patient 1 prior to the initial surgery, clear language errors were observed (Figure 4, Table 1). Overall, 750 left-hemispheric stimulations elicited 119 errors (=all errors), which is equal to an error rate of 15.9%. With regard to the group of no response and performance errors, the highest error rate was observed after stimulation to mPrG, followed by aSMG and pMFG (Figure 4).

#### Intraoperative DCS mapping

VPrG was the only region determined as language-positive during intraoperative DCS mapping but has to be rated as impairment of the primary motor representation of speech rather than real language disturbance, which explains the lacking concordance with rTMS [20,21]. Figure 5 provides an overview on the distribution of language-positive DCS spots.

### Patient 1 – second mapping

#### Preoperative rTMS

During the second mapping session, 390 stimulations of the left hemisphere elicited 62 errors (=all errors), which is an error rate of 15.9% and therefore identical to the first mapping (Table 1). Taking only into account the group of no responses and performance errors, high error rates were elicited by stimulation to mPrG, aSTG, mSTG, dPoG, and opIFG (Figure 4).

#### Intraoperative DCS mapping

This time, vPrG and opIFG were considered language-positive during intraoperative DCS mapping. Figure 5 gives an overview on the distribution of language-positive DCS spots. In this patient, rTMS was able to show us increased activation of the opIFG prior to the second surgery and therefore changes in language organization.

### Patient 2 – first mapping

#### Preoperative rTMS

A number of 675 left-hemispheric stimulations elicited 151 errors (=all errors) of different categories, which is equal to an error rate of 22.4% (Table 1). In the group of no response and performance errors, high error rates were observed after stimulation to aSMG, vPrG, anG, mSTG, and pMFG.

#### Intraoperative DCS mapping

During intraoperative DCS mapping, vPrG was the only language-positive site. However, this time, rTMS was able to elicit no response and performance errors at this region. Again, Figure 5 outlines the distribution of language-positive DCS spots.

### Patient 2 – second mapping

#### Preoperative rTMS

Overall, 405 left-hemispheric stimulations elicited 135 errors (=all errors) in total. This is equal to an error rate of 33.3%. With regard to the initial rTMS session, there was a higher error rate in the second mapping (Table 1). Many regions were prone to errors. In the group of no response and performance errors, high error rates were mainly observed after stimulation to trIFG, vPoG, pSTG, dPoG, mPrG, and dPrG (Figure 4).

#### Intraoperative DCS mapping

VPrG and trIFG were both determined as language-positive during intraoperative DCS mapping. The distribution of language-positive DCS spots is illustrated in Figure 5. Again, rTMS was able to show us language reorganization by additional involvement of trIFG in language processing which was revealed by rTMS due to a severely increased error rate (Figure 4).

### Patient 3 – first mapping

#### Preoperative rTMS

Out of 641 left-hemispheric stimulations 151 elicited errors (=all errors), which is equal to an error rate of 23.6%. With regard to the group of no response and performance errors, the highest error rates were observed after stimulation to pMFG, aSMG, and mSTG (Figure 4).

#### Intraoperative DCS mapping

PMFG and opIFG were primarily affected by DCS during intraoperative language mapping. Figure 5 gives an overview on the distribution of language-positive DCS spots.

### Patient 3 – second mapping

#### Preoperative rTMS

Overall, 258 stimulations of the left hemisphere elicited 83 errors (=all errors), which is equal to an error rate of 32.2%. With regard to the results of the initial rTMS session, we observed a higher error rate during the second mapping (Table 1). Overall, many regions were prone to errors. In the group of no response and performance errors, high error rates were elicited by stimulation to pSTG, dPrG, dPoG, opIFG, and vPrG (Figure 4).

#### Intraoperative DCS mapping

Again, pMFG and opIFG were considered language-positive during intraoperative DCS mapping. An overview on the distribution of language-positive DCS spots during awake surgery is provided by Figure 5. However, although DCS mapping remained stable, rTMS showed increased activation of many CPS regions, which were within the craniotomy but did not show any language impairment by DCS such as vPoG, opIFG, aSMG, and vPrG.

### Patient outcome

As well as in the initial surgery, the second intervention achieved a complete tumor resection in patients 1 and 2 (Figure 1, Table 1). In patient 3, an acute subdural hemorrhage was diagnosed in the postoperative MRI after the first awake surgery combined with increased postoperative aphasia, which resolved completely after immediate hematoma evacuation (Figure 1, Table 1). However, this postoperative MRI also showed a complete tumor resection, whereas the second surgery just achieved a partial resection due to preservation of language function. Concerning postoperative language evaluation, there was no surgery-related increase of aphasia during long-term follow-up in any patient (Table 1).

## Discussion

In patient 1, initial DCS approved vPrG to be a language-positive site, whereas DCS during the second awake surgery determined the vPrG and opIFG to be language eloquent spots. In contrast, rTMS elicited the highest error rates with regard to the combined group of no responses and performance errors by stimulation to mPrG during the first and second mapping (Figures 4 and 5).

In patient 2, initial DCS determined vPrG to be a language eloquent spot, whereas vPrG and trIFG were language-positive sites during the second surgery’s DCS. These findings correlate well with the results of rTMS prior to the first surgery, which showed a high error rate by stimulation to vPrG. In addition, there is a partially good correlation between DCS and rTMS prior to re-resection, when both methods elicited errors by stimulation to trIFG. During rTMS, vPrG was not prone to errors this time (Figures 4 and 5).

In patient 3, it turns out that the opIFG and pMFG were sites with clear language errors during DCS in both awake surgeries. With regard to the combined group of no responses and performance errors, rTMS elicited high error rates by stimulation to pMFG in the first and opIFG in the second mapping (Figures 4 and 5).

Looking at patient 1 and 2, it becomes obvious that initial DCS results and findings during the second awake surgery only correlate partially. In comparison with initial findings, DCS approved an additional CPS region to be language eloquent during re-resection in both cases (Figure 5). This aspect can be suggested to be an expression of brain plasticity for language tasks as reported in other studies [4,22-25]. Additionally, as a result of brain plasticity, rTMS prior to and DCS results during initial surgery only partially corresponded with rTMS and DCS findings of the second examination (Figures 4 and 5). However, rTMS was able to reveal changes in language organization in both cases.

Regarding the rTMS results of those sites included in the craniotomy and undergoing DCS, it appears that stimulations in the precentral gyrus are associated with a high error rate in general which has to be considered as dysarthria by temporarily disturbed function of the motor component of speech rather than actual language impairment [20,21,26].

Moreover, we observed in this cohort, that correlation of rTMS and DCS is much better in anterior language areas compared to posterior sites such as aSMG, pSMG, anG, and temporal sites, which is in accordance with a recently published study [11]. However, we have to keep in mind that the observations in this study cannot be based on sufficient statistics due to the small number of patients.

Comparing the results of all initial rTMS sessions with the corresponding remappings, the error rate was higher during the second mapping in two cases (Figure 4, Table 1). In fact, this primarily outlines the progression of aphasia caused by the growth of the tumor and increasing perifocal edema. With regard to the total amount of stimulations, the rTMS mapping session before initial surgery was performed with a higher number of stimulations than the second one in all three cases (Table 1). This is most likely caused by the learning effect of the examiner but also a sign for the reduced ability to focus of our brain tumor patients when suffering from recurrent glioma.

Moreover, with regard to the extent of resection (Figure 1) many rTMS-positive CPS regions were part of the resection cavity with no long-term impairment of language function. This observation of false-positive rTMS sites can be explained by two facts: on the one hand, language was shown to be organized in a complex network, which can undergo substantial reorganization [3,4,22-24,27]. On the other hand, the induced current density and direction by rTMS differs from that induced by DCS. As it was already described for motor mapping, DCS activates cortical axons directly whereas rTMS activates neurons mainly through indirect intracortical pathways [28-31]. Such unspecific activation or inhibition of these intracortical pathways might identify sites rTMS-positive, which do not carry really essential language function. These differences have to be considered when analyzing rTMS mapping results and the correlation between both methods. To face such potential limitations further studies have to be performed on the optimal rTMS intensity, frequency, and duration to improve our current protocol. Moreover, the immediate effect of rTMS on the cortical excitability requires further profound investigation. The current protocol was used in this study because sufficient results were reported in the past and were also able to induce language errors in all patients [7,10,11]. Moreover, the (by our definition) “false” positive rTMS sites may also identify language sites, which are not intraoperatively defined and in this case may also be regarded as potentially dangerous areas for resection. The CPS areas, although relatively small, still exceed the size of the 10-mm error margin of the DCS, and thus some false positive results may be due to less dense spatial sampling by the DCS [1,32].

Pain during rTMS sessions was measured for each patient as mentioned before. In general, language mapping was tolerated well by all subjects, which is shown by the fact that there was no abandonment due to stimulation-related discomfort like in other studies published before (Table 1) [7,10].

Concerning the value of this new tool, we also have to keep in mind that additional preoperative information on the distribution of language eloquent cortical regions would also enable tailored craniotomies for a more targeted intraoperative DCS. However, our results also show that with the current protocol rTMS is more applicable to show language reorganization instead of language-eloquent cortical sites per se.

Concerning fMRI as another non-invasive method, a recently published case report shows that fMRI failed to provide adequate accuracy compared to DCS and rTMS [10]. However, fMRI cannot be principally regarded as less accurate than DCS or rTMS but there are many studies at hand, which proved insufficient accordance of fMRI to DCS [5,10,33,34]. Yet, when comparing lesion-based investigations by rTMS with hemodynamic studies such as fMRI, one should remember that fMRI does not show neurological activation per se but changed oxygenation levels within the brain which can also be based on impaired tissue oxygenation by the adjacent intracerebral lesions operated on in this cohort [5,10,33-35].

### Future impact of nTMS on neurosurgery

Preoperative nTMS for motor mapping allows us today to inform each patient individually of possible transient postoperative paresis, as we know exactly how close the rolandic region is to the intended resection border in every single case. Thus, we are able to assess operative risks for permanent paresis more precisely and we can use these data to prepare the patient preoperatively.

Hence, rTMS data for language mapping have also influence that cannot be measured by simple outcome studies but may lead to better prepared patients and thus improve patients’ satisfaction. Likewise, rTMS language mapping could allow us to consider indication for surgery by outlining language negative regions quite reliably. Thus, if we are able to further improve the precision and reliability of this method, we might even be able to operate some of these patients without awake surgery.

However, with regard to the low specificity and PPV of this method, we need to improve rTMS language mapping significantly before thinking about further applications.

## Conclusions

This study demonstrates that rTMS is able to partially detect language negative regions prior to awake surgery and as a tool during routine follow-up of such patients with recurrent glioma. As patients with glioma frequently suffer from recurrence, it is important to have a reliable preoperative technique to detect eloquent sites non-invasively to evaluate eligibility for repetitive surgery and for providing useful functional data for the upcoming awake procedure. Knowledge of stability or any plasticity of language dominant brain regions is crucial for the evaluation of surgical options and the introduction of a reliable technique would support new approaches for glioma surgeons [22-24]. However, with the current protocol, rTMS is still not accurate enough to turn down awake surgery and DCS during awake surgery still remains gold standard for the resection of language eloquent tumors.

## Abbreviations

CPS: Cortical parcellation system; DCS: Direct cortical stimulation; fMRI: Functional magnetic resonance imaging; nTMS: Navigated transcranial magnetic stimulation; rTMS: Repetitive navigated transcranial magnetic stimulation; VAS: Visual analogue scale; WHO: World Health Organization

## Competing interests

The authors declare that they have no competing interests. The study was completely financed by institutional grants from the Department of Neurosurgery and the authors declare that they have no conflict of interest affecting this study.

## Authors’ contributions

SK is responsible for the original idea, the concept, design, data acquisition and statistical analyses. SK performed literature research and drafted the manuscript. NS was responsible for data acquisition, handled the acquired data, performed literature research and drafted the manuscript. TH and SI were responsible for data acquisition. BM approved and corrected the final version of the manuscript. FR revised the manuscript, approved and corrected the final version. All authors read and approved the final manuscript.

## Authors’ information

NS, TH, and SI are medical student who are performing a considerable number of rTMS studies. All other authors are strongly involved in the treatment of brain tumors including awake surgery, preoperative mapping, and intraoperative neuromonitoring in a specialized neurooncological center. BM is chairman and FR is vice chairman of the department.
